# Persistent Risk of Pulmonary Embolism in Acute Pancreatitis Despite Prophylactic Anticoagulation

**DOI:** 10.7759/cureus.74249

**Published:** 2024-11-22

**Authors:** Talal Alomar, Anupama Somaratna, Deepti Boddupalli

**Affiliations:** 1 Internal Medicine, Creighton University School of Medicine, Phoenix, USA

**Keywords:** acute inflammation causing pulmonary embolism, massive pulmonary embolism, pancreatitis, pancreatitis and pulmonary embolism, pulmonary embolism despite anticoagulation

## Abstract

Acute pancreatitis, a sudden inflammatory condition, can lead to a hypercoagulable state resulting in complications such as deep vein thrombosis (DVT) or pulmonary embolism (PE). This case report discusses a unique presentation of a massive PE in a patient with acute pancreatitis despite being on appropriate prophylactic anticoagulation.

A 27-year-old man presented with acute abdominal pain, nausea, and vomiting. He was diagnosed with diabetic ketoacidosis (DKA) and acute pancreatitis and admitted to the ICU. He was treated with prophylactic enoxaparin. On day 16, he experienced acute respiratory decompensation, and CT angiography revealed bilateral PEs, including a right main pulmonary artery saddle embolus. The patient underwent emergent thrombectomy with the immediate resolution of symptoms. He was transitioned to therapeutic heparin and later discharged on apixaban. A two-month follow-up showed no recurrence of PE.

This case underscores the critical need to consider PE in patients with inflammatory conditions, even when on prophylactic anticoagulation. The hypercoagulable state induced by pancreatitis can overcome standard anticoagulation measures, leading to severe complications. Current guidelines may not adequately address the anticoagulation needs in such inflammatory states. Therefore, weight-based dosing of anticoagulants should be considered for patients with significant inflammation. This report highlights the necessity for vigilance in monitoring for PE in similar clinical scenarios to improve patient outcomes and inform future guidelines.

## Introduction

Acute pancreatitis is a sudden inflammatory condition caused by gallstones, alcohol, hypertriglyceridemia, and hypercalcemia or which can be idiopathic [1]. Pancreatic swelling leads to the blockage of enzymatic drainage into the duodenum, leading to autodigestion and inflammation. The inflammatory response causes a hypercoagulable state that can lead to deep vein thrombosis (DVT) or pulmonary embolism (PE) [2]. A PE is a blockage of the main artery of the lung or one of its branches by a blood clot that causes dyspnea, chest pain, and hypoxia due to severe ventilation-perfusion mismatch [3]. If left untreated, the mortality rate for PE can be as high as 30% [4]. There are no accurate statistics as to the rate of PE in acute pancreatitis given that it is so rare.

Many minimally or non-ambulatory patients in the hospital are placed on DVT prophylaxis with anticoagulants. In rare circumstances, a massive PE can develop such as what happened to our patient.

## Case presentation

The patient is a 27-year-old man with a body mass index of 29 who presented to the emergency department with a two-day history of abdominal pain, nausea, and vomiting. Labs on admission showed elevated white blood cell count, glucose, urine ketones, triglycerides, and lipase (Table 1). Abdominal CT scan revealed acute pancreatitis likely secondary to uncontrolled diabetes and hypertriglyceridemia. The patient was admitted to the ICU for diabetic ketoacidosis (DKA) in the setting of undiagnosed diabetes mellitus. He was downgraded to the floor on day 5 and had an abdominal drain placed on day 7 after he became septic secondary to an imaging-confirmed peripancreatic abscess. Based on the Atlanta criteria, the patient had moderately severe acute pancreatitis. Peritoneal fluid cultures did not show the growth of any organisms. At this time, the patient was clinically stable and DKA had resolved. He was ambulating in hallways without chest pain or tachycardia and tolerating diet without evidence of diarrhea or abdominal pain.

**Table 1:** Pertinent labs on admission Table of pertinent lab results on admission. Elevated values are in bold.

Lab test	Result	Reference range
White blood cells	19.7 thousand/uL	3.6-11.1 thousand/uL
Blood glucose	364 mg/dL	65-99 mg/dL
Lipase	811 U/L	0-160 U/L
Creatinine	1.55 mg/dL	0.65-1.25 mg/dL
Lactic acid	4.1 mmol/L	0.4-2.2 mmol/L
Potassium	4.6 mmol/L	4.1-5.3 mmol/L
Triglycerides	8177 mg/dL	<150 mg/dL
Urine ketones	80 mmol/L	<0.6 mmol/L

**Figure 1:**
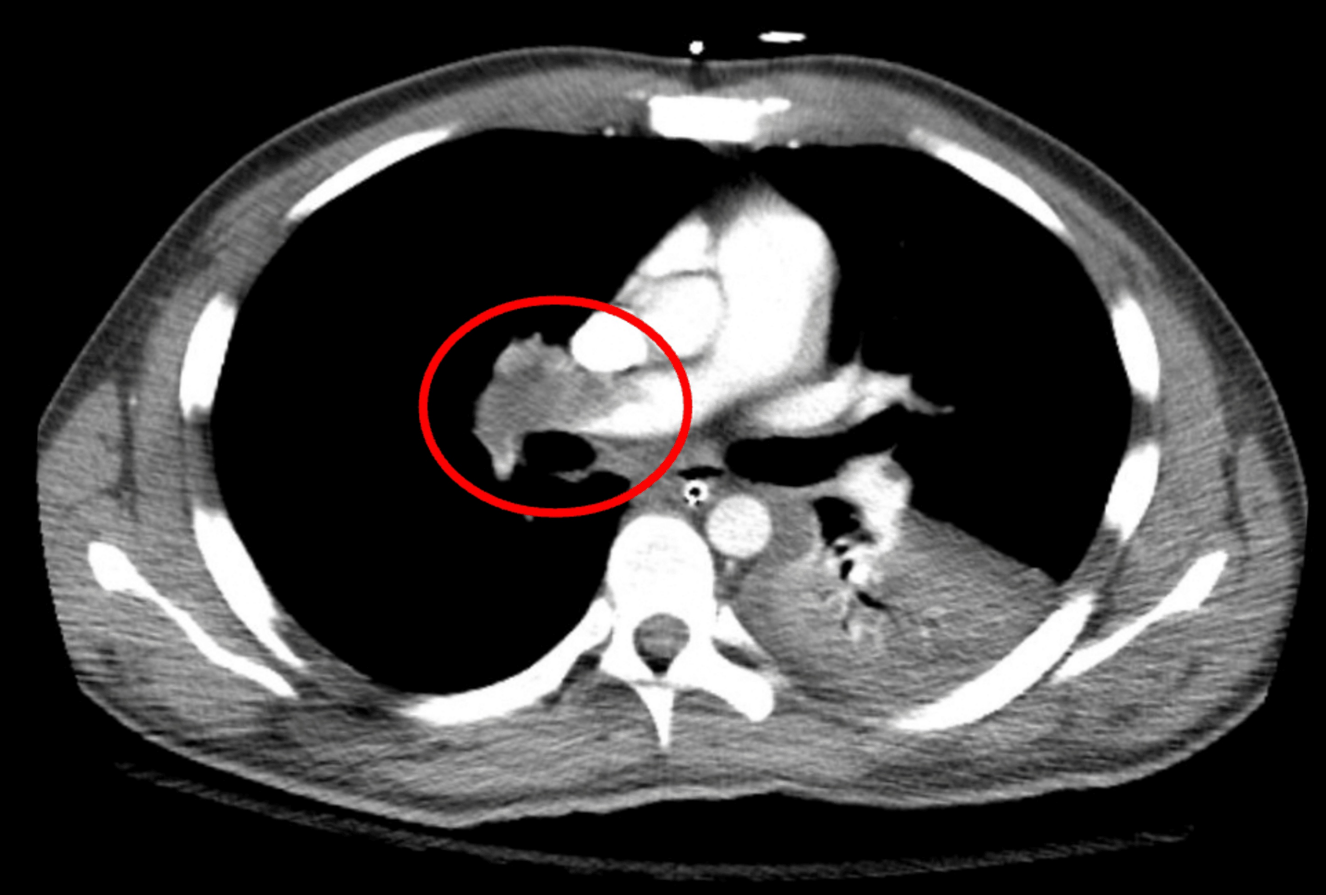
CT angiogram CT angiography of the chest showing large pulmonary emboli in the right pulmonary artery branch. The embolism is circled in red.

**Figure 2:**
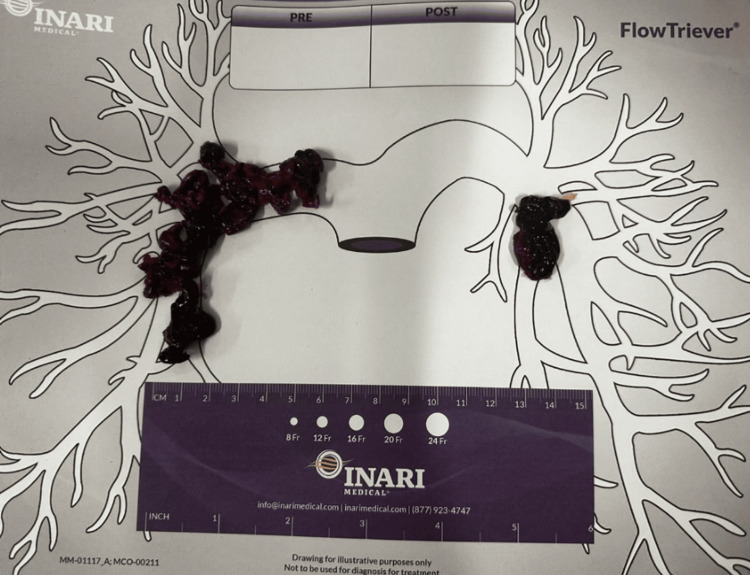
Image of the significant pulmonary emboli removed with thrombectomy

On day 16, the patient had an acute decompensation requiring a high-flow nasal cannula with a fraction of inspired oxygen (FiO2) of 95% and a 50 L flow rate. He was intubated for acute hypoxic respiratory failure. Bedside chest X-ray showed pleural effusion and increased perihilar congestion. CT angiography demonstrated bilateral PEs, including right main pulmonary artery saddle embolus (Figure 1). The PE was not present on his CT chest done the week prior. The patient underwent emergent thrombectomy with interventional radiology resulting in the immediate resolution of symptoms (Figure 2). Upon further questioning, he reported no family history of blood clots, recent travels, or trauma. Testing for the most common thrombophilias such as protein C and S deficiency as well as factor V Leiden mutation was negative. A CT of the abdomen was done after the episode to assess for splenic vein thrombosis or other clots and was negative. The patient was on a standard prophylactic enoxaparin dose of 40 mg during his entire hospital stay leading up to the PE. After the thrombectomy, he was transitioned to therapeutic heparin with a partial thromboplastin time (PTT) between 60 and 100 seconds until his discharge 10 days later. The patient was discharged on appropriate antibiotics and apixaban, per guidelines on post-PE treatment. On the two-month follow-up, he had no recurrence of PE.

## Discussion

Despite our patient being on appropriate prophylactic dosing of enoxaparin, he still developed a massive PE that required surgical intervention. It appears that the pro-inflammatory state of pancreatitis was able to overcome the anticoagulation. Virchow's triad states that blood clots form from stasis, endothelial dysfunction, and hypercoagulable states like infection or genetic mutations [5]. Our patient had blood flow stasis from a long ICU stay and decreased ambulation and had significant inflammation from the pancreatitis with an abscess. Current theories suggest that pancreatic cysts connected to the pancreatic duct may infiltrate blood vessels, releasing pancreatic enzymes that damage the vascular endothelium, thus triggering the release of procoagulant substances like tissue factor and cytokines, leading to a hypercoagulable state [6]. Additionally, systemic inflammatory response syndrome (SIRS) often accompanies acute pancreatitis, with inflammatory mediators further damaging the vascular endothelium. These mechanisms are similar to those of cancer patients, who also have increased clotting due to inflammatory mediators [7,8]. 

The efficacy of enoxaparin at different doses and in comparison to other medications has been extensively studied. In their prospective trial to evaluate the efficacy of enoxaparin at various doses, Spiro et al. [9] report that 14% of patients on enoxaparin 40 mg developed a DVT, significantly less than a 10 mg dose. Our patient was on 40 mg of enoxaparin, the standard prophylactic dose. Enoxaparin is significantly more effective than heparin and lowers the risk of DVT by 74% compared to heparin [10]. Direct factor Xa inhibitors like rivaroxaban were found to have equal efficacy as enoxaparin [11]. So, enoxaparin was an appropriate anticoagulant choice, but the dosing may have been too low in the setting of acute inflammatory processes. Current guidelines only recommend increasing the dose of enoxaparin for patients with a body mass index above 40, so our patient did not meet this requirement [12]. There are currently no guidelines on anticoagulation adjustments in acute pancreatitis or severe inflammatory conditions; however, the following trials suggest anticoagulation should be increased. Zwicker et al. [13] conducted a randomized controlled trial to see if weight-based enoxaparin prophylaxis in cancer patients could prevent DVTs. They found that the control group given 40 mg of enoxaparin had a 22% DVT rate, while the treatment group that was given a weight-based enoxaparin dose of 1 mg/kg had a 5.9% incidence of DVT, a statistically significant reduction. The weight-based dosing group had no instances of hemorrhage. Abdulla et al. [14] found that only 33% of critically ill patients on standard prophylactic dosing of enoxaparin had goal anti-Xa levels. So, weight-based enoxaparin dosing for patients with severe pro-inflammatory conditions like malignancies or severe pancreatitis should be considered. Anti-Xa levels should also be checked in these patients to confirm appropriate anticoagulation. 

The symptoms of PE, which include difficulty breathing, chest pain, and palpitations, vary depending on the size and number of emboli, the rate of embolization, the location of the embolism, and the patient's cardiopulmonary function [3]. Clinical signs such as hypoxia, cyanosis, tachypnea, and tachycardia can help in the diagnosis. Early recognition of PE is crucial because it is a potentially life-threatening condition where timely intervention can prevent severe complications such as right ventricular failure and sudden death. CT angiography is the preferred diagnostic imaging to visualize the pulmonary vasculature. Patients with PE who are hemodynamically stable can be treated with heparin which will prevent further coagulation and lead to the dissolution of the clot with time. In cases where large PEs cause hemodynamic instability, such as with our patient, treatment with percutaneous thrombectomy is preferred [3]. 

## Conclusions

To our knowledge, this is the first case of a massive PE in the setting of acute pancreatitis, despite the patient being on guideline-directed treatment. Other cases of PE in acute pancreatitis involved patients who were not on anticoagulation. It is essential for physicians to keep PE on the differential for patients with any inflammatory conditions whether it be autoimmune conditions, pancreatitis, or malignancy. Prompt treatment is key for survival. Physicians should consider weight-based DVT prophylaxis and monitoring anti-Xa levels in patients with high levels of inflammation. Further research on appropriate anticoagulation in cases of extreme inflammation is needed.
